# Does anticholinergic medication use on presentation to a rural memory clinic predict cognitive or functional decline over one year?

**DOI:** 10.1177/13872877261424232

**Published:** 2026-03-09

**Authors:** Devin Proulx, Andrew Kirk, Megan O'Connell, Debra Morgan

**Affiliations:** 1 College of Medicine, 7235 University of Saskatchewan, Saskatoon, SK, Canada; 2 Department of Neurology, 7235 University of Saskatchewan, Saskatoon, SK, Canada; 3 Department of Psychology & Health Studies, 7235 University of Saskatchewan, Saskatoon, SK, Canada; 4 Canadian Centre for Rural and Agricultural Health, 7235 University of Saskatchewan, Saskatoon, SK, Canada

**Keywords:** acetylcholine, Alzheimer's disease, cholinergic agents, cholinesterase, dementia

## Abstract

**Background:**

Anticholinergic medications carry increased risk of worsening cognition, particularly in patients with dementia.

**Objective:**

Cognitive and functional scores of patients were compared at 1-year follow up after cessation of prior anticholinergic medications to those who were not taking anticholinergics at baseline to assess whether prior anticholinergic use affected dementia prognosis in memory clinic patients.

**Methods:**

Longitudinal data from 578 consecutive patients with diagnoses including Alzheimer's disease (AD), frontotemporal and vascular dementia, Lewy body dementia, mild cognitive impairment and dementia due to non-AD etiologies compared patients taking anticholinergic drugs to those taking none at intake. Anticholinergic drugs were discontinued in all patients at initial visit. Mini-Mental Status Examination (MMSE) and Functional Activities Questionnaire (FAQ) were administered at intake and 1-year follow up.

**Results:**

Between the no-anticholinergic and anticholinergic groups, MMSE at baseline did not significantly differ when the entire sample was compared. In addition, MMSE score change between baseline and follow-up did not significantly differ. Similarly, no significant differences were observed between groups in FAQ at baseline or in FAQ change between timepoints. Subgroup analysis of only those with AD yielded statistically significant differences in initial MMSE. However, these groups had statistically similar follow-up MMSE. Although initial FAQ scores were similar, there were significant differences in follow-up FAQ in patients with AD.

**Conclusions:**

Findings suggest that patients with AD presenting on anticholinergic medications may do worse cognitively and functionally than those who were never on anticholinergics despite baseline discontinuation. Anticholinergic medication use in older adults should be approached cautiously.

## Introduction

Cholinesterase inhibitors remain a mainstay in dementia treatment, resulting in clinically significant improvement in cognition and reduced mortality.^1,2^ In Alzheimer's disease (AD), it is understood that acetylcholine receptors and their degenerative pathophysiology play an important role in the onset and progression of the disease. Specifically, in AD, degeneration of cholinergic neurons in the nucleus basalis of Meynert contributes to progressive impairment in memory and cognition via reduced pre and postsynaptic acetylcholine.^3^ The relationship between cholinergic activity and AD has been investigated previously, with studies demonstrating a significant improvement in AD symptoms when patients are treated with cholinesterase inhibitors, and the inverse effect on memory is seen in patients who are treated for other illnesses with anticholinergic medications.^3^

While it is known that anticholinergic drugs can worsen cognition,^4^ no direct mechanistic link has been made between dementia progression and anticholinergic treatment in patients with dementia. At least a fifth of all patients diagnosed with dementia or other cognitive impairments are taking anticholinergic drugs, and those on anticholinergic drugs have poorer Mini-Mental State Examination (MMSE)^5^ scores than patients not taking anticholinergic medications.^4,6^

Current literature describing the effects of anticholinergic medication on dementia progression reveals mixed results. A recent systematic review found a positive and dose-dependent relationship between anticholinergic drug use and dementia risk. Zheng et al. further states that effects of anticholinergic medications on dementia risk is categorical in nature, that antiparkinsonian, urological, and antidepressant drugs enhance the risk of dementia, while cardiovascular and gastrointestinal drugs may play protective roles in dementia risk.^7^ A retrospective cohort study found significant negative changes in MMSE scores between patients already diagnosed with dementia on high and low dose anticholinergic drugs, noting that patients with a high anticholinergic burden had their MMSE scores decline more quickly than those patients with a low anticholinergic burden.^8^ Bishara et al. saw a significant increase in all-cause mortality for those patients with high anticholinergic burden.^8^

In contrast, Gildeners et al., in a 2023 publication found that while anticholinergic medications were a significant risk factor for developing mild cognitive impairment, increased risk of developing dementia was not present in older adults treated with anticholinergic medications.^9^ Dyer et al. discussed how anticholinergic use negatively affected progression of some cognitive measures but not others in patients with mild to moderate dementia, with dementia progression being particularly worse in those patients with the *APOE* ε4 allele.^10^ A systematic review investigating the association between anticholinergic medications and clinical outcomes in patients with pre-existing dementia found mixed results relating to negative cognitive outcomes but, similar to previous studies, found a marked increase in all-cause mortality in patients diagnosed with dementia who were treated with anticholinergic medications.^11^

This retrospective study aimed to explore the association of anticholinergic treatment with outcomes across various cognitive and functional measures such as the MMSE,^12^ Global Deterioration Scale Rating (GDSR)^13^ and the Functional Activities Questionnaire (FAQ)^14^ in patients presenting with cognitive symptoms. The MMSE acts as a screening tool for cognitive impairment and has a score ranging from 0–30. Scores on the MMSE below 25 are considered mild, scores under 20 are considered moderate and scores under 10 are considered severe. The GDSR is 7 stage measurement tool that tracks progression of neurocognitive degenerative diseases like AD, ranging from no measurable cognitive decline to very severe. The FAQ is another measurement tool utilized to assess a patient's independence with instrumental activities of daily living. FAQ scores are often collected via a collateral source to ensure reliability. The FAQ is scored from 0–30, with scores over 5 signifying possible cognitive functional impairment, and scores over 9 signifying impaired cognitive function. Often These metrics were assessed at initial presentation to the University of Saskatchewan's Rural and Remote Memory Clinic (RRMC) and at 1-year follow-up. These findings were compared with patients who presented to the RRMC and were not taking anticholinergic drugs at the time of first visit to assess whether there is a link between prior anticholinergic drug use and cognitive or functional decline.

## Methods

578 consecutively assessed patients presented to the RRMC between 2004 and 2020 were included in the current study. At first visit, patient demographic information was obtained as well as current medication lists and medical diagnoses. Anticholinergic medications were always discontinued at the first RRMC visit. Patient's cognition and function was measured via relevant scales (MMSE, FAQ, GDSR) at presentation and at 1-year follow-up. Patient data for this study was collected from patient records and organized in a deidentified list for analysis. We defined a set of anticholinergic medications with notable anticholinergic burden based on the American Geriatrics Society 2023 updated AGS Beers Criteria.^15^ A table of all the anticholinergic medications considered in this analysis were included in Table 1.

**Table 1.** Medications with anticholinergic burden considered in research.

Amitriptyline (Elavil)	Chlorpromazine (Thorazine)	Dicyclomine (Bentyl)	Hyoscyamine (Anaspaz, Levbid, Levsin, Levsinex, NuLev)	Olanzapine (Zyprexa)	Propantheline	Trihexyphenidyl
Amoxapine	Clomipramine (Anafranil)	Dimenhydrinate (Dramamine)	Imipramine (Tofranil)	Orphenadrine (Norflex)	Pseudoephedrine HCl/Triprolidine HCl (Aprodine)	Trimipramine (Surmontil)
Atropine	Clidinium-chlordiazepoxide (Librax)	Diphenhydramine	Loperamide	Oxybutynin (Ditropan, Oxytrol)	Ranitidine	Triprolidine
Benztropine (Cogentin)	Clozapine (Clozaril)	Disopyramide (Norpace, Norpace CR)	Loratadine	Paroxetine (Brisdelle, Paxil)	Loratadine	Trospium
Belladonna alkaloids	Cyclobenzaprine (Amrix, Fexmid, Flexeril)	Doxepin (Adapin, Silenor, Sinequan)	Loxapine	Perphenazine (Trilafon)	Scopolamine	
Brompheniramine	Cyproheptadine (Periactin)	Doxylamine (Unisom)	Meclizine (Antivert, Bonine)	Prochlorperazine (Compazine)	Solifenacin (Vesicare)	
Carbinoxamine (Arbinoxa, Karbinal ER, Palgic)	Darifenacin (Enablex)	Fesoterodine (Toviaz)	Methocarbamol (Robaxin)	Prochlorperazone	Thioridazine (Mellaril)	
Clemastine	Desipramine (Norpramin)	Flavoxate	Methscopolamine (Pamine, Pamine Forte)	Promethazine (Phenergan)	Tolterodine (Detrol)	
Chlorpheniramine	Dexchlorpheniramine (Ala-Hist IR)	Hydroxyzine (Atarax, Vistaril)	Nortriptyline (Pamelor)	Protriptyline (Vivactil)	Trifluoperazine (Stelazine)	

Statistical analysis of the data was done using SPSS software. Patient's RRMC admission MMSE and FAQ scores were compared to their year-1 follow-up MMSE and FAQ scores. Further analyses were completed to compare possible statistical differences in patients with different forms of dementia. The project was approved by the University of Saskatchewan Biomedical Ethics Committee. Data safety compliance was ensured via their protocol.

## Results

Data from 578 consecutively assessed patients were analyzed (M age 70.34; SD = 11.56; 60% female). AD was the most frequent diagnosis (n = 212; 37%), 20% (n = 117) were diagnosed with dementia due to non-AD diagnoses which included vascular dementia, frontotemporal dementia, Lewy body dementia, and Parkinson's dementia. Approximately 15% of patients were diagnosed with mild cognitive impairment (n = 84), 4% (n = 24) were diagnosed with cognitive impairment due to another etiology (e.g., lifelong cognitive impairment), and 24% (n = 141) were diagnosed with subjective cognitive impairment. 127 patients were taking at least 1 anticholinergic medication at presentation

When initial MMSE was compared across all diagnoses between patients taking and those not taking anticholinergics at presentation (Mnone = 23.94; SD = 4.56 versus Mantichol = 23.13; SD = 5.80; t504 = 0.82, p = 0.08, d = 0.17) they were similar. In addition, the difference between baseline MMSE and MMSE at 1 year follow-up (delta MMSE Mnone = −0.54; SD = 2.70 versus Mantichol = −0.49; SD = 2.45; t157 = 0.09, p = 0.93, d = 0.02) was not statistically significant in either group across all diagnoses. The initial FAQ and change in FAQ were statically similar for both cholinergic groups (initial Mnone = 12.74; SD = 8.50 versus Mantichol = 12.18; SD = 9.11; t283 = 0.46, p = 0.32, d = 0.06; change at 1-year-follow-up Mnone = −0.90; SD = 4.23 versus Mantichol = −0.29; SD = 4.71; t112 = −0.64, p = 0.52, d = 0.14).

When the group diagnosed with dementia due to AD were analyzed separately, however, there were statistically significant but small differences in initial MMSE (Mnone = 21.11; SD = 4.10 versus Mantichol = 19.57; SD = 5.41; t187 = 1.9, p = 0.03, d = 0.35), but they had statistically similar 1 year follow-up MMSE scores (Mnone = 21.28; SD = 4.72 versus Mantichol = 22.73; SD = 4.74; t53 = −1.02, p = 0.16, d = 0.31) and the change in MMSE scores were similar for both groups. Although the initial FAQ scores were similar, there were significant differences in the FAQ at one year follow-up (Mnone = −13.48; SD = 8.30 versus Mantichol = −17.86; SD = 8.86; t54 = −1.68, p = 0.05, d = 0.52). Due limited data analyses segmented by cholinesterase inhibitors and effects on MMSE and FAQ outcomes could not be completed.

## Discussion

This study resulted in a few key findings. Firstly, in aggregate analysis there was no statistically significant difference in MMSE scores from patients taking anticholinergic medications versus those who were not upon admission to the RRMC compared to one year follow-up. However, when broken down by dementia diagnosis there seems to be a small measurable difference in the AD cohort of patients presenting on anticholinergic medications versus those who were not. Initial anticholinergic treated AD cohort MMSE scores differed minimally from the aggregate study cohort and there was a significant difference in the anticholinergic treated AD cohort's follow-up FAQ scores compared to initial FAQ scores with those who were not treated with anticholinergic medications.

Some explanations for these findings could be in the demographic differences between cohorts. As seen in Table 2, the AD cohort (N = 212) had the second oldest average age at 74.5 behind only those with Lewy body dementia (N = 28) at average age of 78.2. However, the Alzheimer's cohort represented a much larger proportion of the sample. There could be a possible connection between the cognitive burden of anticholinergic medications on older patients compared to younger ones, however this remains to be demonstrated in current literature. Furthermore, there could be an explanation for differences in this study's measured outcomes based on the etiology of the dementia and the organic illness course progression expected with each diagnosis.

**Table 2.** Demographics by dementia, mild cognitive impairment or unspecified diagnosis.

Dementia category	Alzheimer's disease	Frontotemporal and vascular dementia	Lewy body dementia	Mild cognitive impairment	Other
Participants N = 578	212	88	28	89	38
AVG Age = 70.34	74.5	68.5	78.2	71.5	65.4
% Female = 60.1	69.6%	46.2%	39.3%	56.1%	31.6%
AVG MMSE = 23.94	20.25	21.4	21.5	26.7	25.4
AVG ATCH Med # = 0.259	0.213	0.208	0.250	0.180	0.370

Interestingly, when comparing cohorts based on the average number of anticholinergic medications taken upon admission to the RRMC, the total cohort's average was higher than the AD cohort (0.259 versus 0.217). When compared with other dementia categories, there appears to be significant variance between groups without a measurable effect on initial and follow-up MMSE or FAQ scores. Therefore, these findings do not suggest a correlation between the number of anticholinergic medications taken and the subsequent effect on cognition. This suggests that there could be other cofounding factors such as drug-drug interactions, specific anticholinergic medication pharmacokinetic effects, or demographic variables unaccounted for. This study may be limited by its sample size as only 578 patients were included. One analytic limitation of this study was the inability to exact a measurable pattern between specific anticholinergic medications and their specific effects on the MMSE and FAQ scores.

Other factors affecting dementia progression in patients were not isolated, potentially leading to a spurious correlation between patients’ anti-cholinergic use and their dementia progression. Lifestyle factors and psychiatric conditions were not isolated in this study but seem to contributory effects on overall dementia progression.^16,17^ For example, some of the medical conditions treated with anticholinergics may have affected dementia prognosis before initial RRMC presentation, limiting the study's ability to capture patients’ anticholinergic treatment effects in totality. Further, no dose dependent relationship between anti-cholinergic use and dementia progression was assessed, which may have contributed to the measured outcome. One last consideration not accounted for in measured outcomes was how drug-drug interactions and medical comorbidities may have affected measured outcomes. Further research into anticholinergic dose dependent and time dependent treatment effects on cognition is necessary.

In conclusion, this study adds to the current body of literature of anticholinergic medication use and its increased risk for cognitive decline in the dementia population. Caution should be exhibited when considering maintaining patients on anticholinergic medications in the setting of endorsed signs of cognitive degeneration and especially early signs of AD.
